# Precision diagnostics in transplanted organs using microarray-assessed gene expression: concepts and technical methods of the Molecular Microscope^®^ Diagnostic System (MMDx)

**DOI:** 10.1042/CS20220530

**Published:** 2024-05-31

**Authors:** Katelynn S. Madill-Thomsen, Philip F. Halloran

**Affiliations:** 1 Department of Medicine, University of Alberta, Edmonton, AB, Canada; 2 Alberta Transplant Applied Genomics Center, University of Alberta, Edmonton, AB, Canada

**Keywords:** biopsy, genomics, kidney, machine learning, molecular diagnostics, transplant

## Abstract

There is a major unmet need for improved accuracy and precision in the assessment of transplant rejection and tissue injury. Diagnoses relying on histologic and visual assessments demonstrate significant variation between expert observers (as represented by low kappa values) and have limited ability to assess many biological processes that produce little histologic changes, for example, acute injury. Consensus rules and guidelines for histologic diagnosis are useful but may have errors. Risks of over- or under-treatment can be serious: many therapies for transplant rejection or primary diseases are expensive and carry risk for significant adverse effects. Improved diagnostic methods could alleviate healthcare costs by reducing treatment errors, increase treatment efficacy, and serve as useful endpoints for clinical trials of new agents that can improve outcomes. Molecular diagnostic assessments using microarrays combined with machine learning algorithms for interpretation have shown promise for increasing diagnostic precision via probabilistic assessments, recalibrating standard of care diagnostic methods, clarifying ambiguous cases, and identifying potentially missed cases of rejection. This review describes the development and application of the Molecular Microscope® Diagnostic System (MMDx), and discusses the history and reasoning behind many common methods, statistical practices, and computational decisions employed to ensure that MMDx scores are as accurate and precise as possible. MMDx provides insights on disease processes and highly reproducible results from a comparatively small amount of tissue and constitutes a general approach that is useful in many areas of medicine, including kidney, heart, lung, and liver transplants, with the possibility of extrapolating lessons for understanding native organ disease states.

## Introduction

This review outlines the development and refinement of the Molecular Microscope® Diagnostic System (MMDx) technology using genome-wide measurements expression in kidney, heart, lung, and liver transplant biopsies [1–23]. MMDx aims to discover the molecular basis of the rejection and parenchymal injury states (‘parenchymal’ referring to elements of the tissue responsible for organ function, plus their supporting matrix and microvasculature, distinct from infiltrating cells) in transplant populations [1–3,8,22,24] and translate these lessons into diagnostic services. MMDx also seeks to define the relationship between the genome-wide molecular phenotype of the transplant and other diagnostic systems such as histology, donor-specific antibody (DSA), and donor-derived cell-free DNA (dd-cfDNA) [25–31]. Some aspects of the MMDx system and its development have previously been described [2,32–36] and recently reviewed [1,2].

In any diagnostic system, establishing the probability that a disease is present involves two steps: (1) measure designated features and (2) interpret feature measurements using predefined algorithms [5]. In MMDx step 1, microarrays assess messenger RNA (mRNA) expression in the biopsy, currently measuring expression of 49,495 probe sets reflecting the expression of 19,462 unique genes [11]. In MMDx step 2, these measurements are interpreted using ensembles of predefined machine-learning derived algorithms and compares the biopsy to a reference set of previously characterized biopsies. MMDx assesses rejection states – T cell-mediated rejection (TCMR) and antibody-mediated rejection (ABMR) – and parenchymal injury states, including recent injury (acute kidney injury [AKI]) and irreversible atrophy-fibrosis. The extent of injury correlates with impaired function and is critical to understanding the risk of graft loss [3,4,8,14].

The MMDx system has been developed over several decades, originating in mouse studies and progressing to large studies of human transplant biopsies. More recently, we have focused on analyses establishing the reproducibility and robustness of the techniques used in MMDx, its relationship to other diagnostic approaches currently in use (e.g., histology, dd-cfDNA), and examining transplant injury separate from rejection.

Once the MMDx-Kidney test was well-defined, the MMDx system was translated into heart, lung, and liver transplants where biopsy assessment is more challenging and where the standard of care diagnoses are often less well-established [37–40]. Molecular diagnostic systems that are rigorously developed, based on precise measurements, interpreted with statistical and machine-learning techniques, and appropriately validated have the potential to address the current unmet needs for accurate assessment that clinicians are requesting to improve patient care. Additionally, genome-wide measurements and molecular profiling data are a critical component of our investigations into post-transplant outcomes, disease mechanisms, and our understanding of response to therapy. Differentially expressed genes show us what is altered by successful treatment, what expression is increased or decreased during rejection episodes or other diseases causing tissue injury, and point us to a more complete picture of the interactions between the transplanted organ and the recipient.

This review will discuss various aspects of the overall MMDx approach, the methodology employed throughout MMDx studies, and the technology development that has led to the current MMDx report system in kidney, heart, lung, and liver transplant populations.

## Probabilities and continuous data in medicine

Medicine has historically focused on categorical data (e.g., diagnoses). Because the final decision to treat or not to treat is often binary, distinct classes and categories of patients are assigned, while acknowledging some uncertainty and complexity around diagnostic thresholds, i.e., borderline cases. For example, a diagnostic report can state a result in terms of grades 0-3, but this fails to recognize samples that were actually on the boundaries of those categories (near cutoffs). Although many standard of care classifications remain categorical, probabilities are becoming more common in medical testing [41] and clinicians can and do use probabilities in their practice [42–44]. Continuous, probabilistic measurements present the advantage of conveying the degree of ‘confidence’ or predicted accuracy of the test results, and can also convey a spectrum of disease severity, therefore better informing dosage decisions. Probabilities, such as those reported by MMDx, provide a continuous value stating how likely it is that the given sample had a specific feature, can designate the degree of activity and the stage, and allow the clinician making treatment decisions to balance their level of confidence in the disease assessment against the cost of under- versus over-diagnosis.

## Machine learning in MMDx

**Figure 1:**
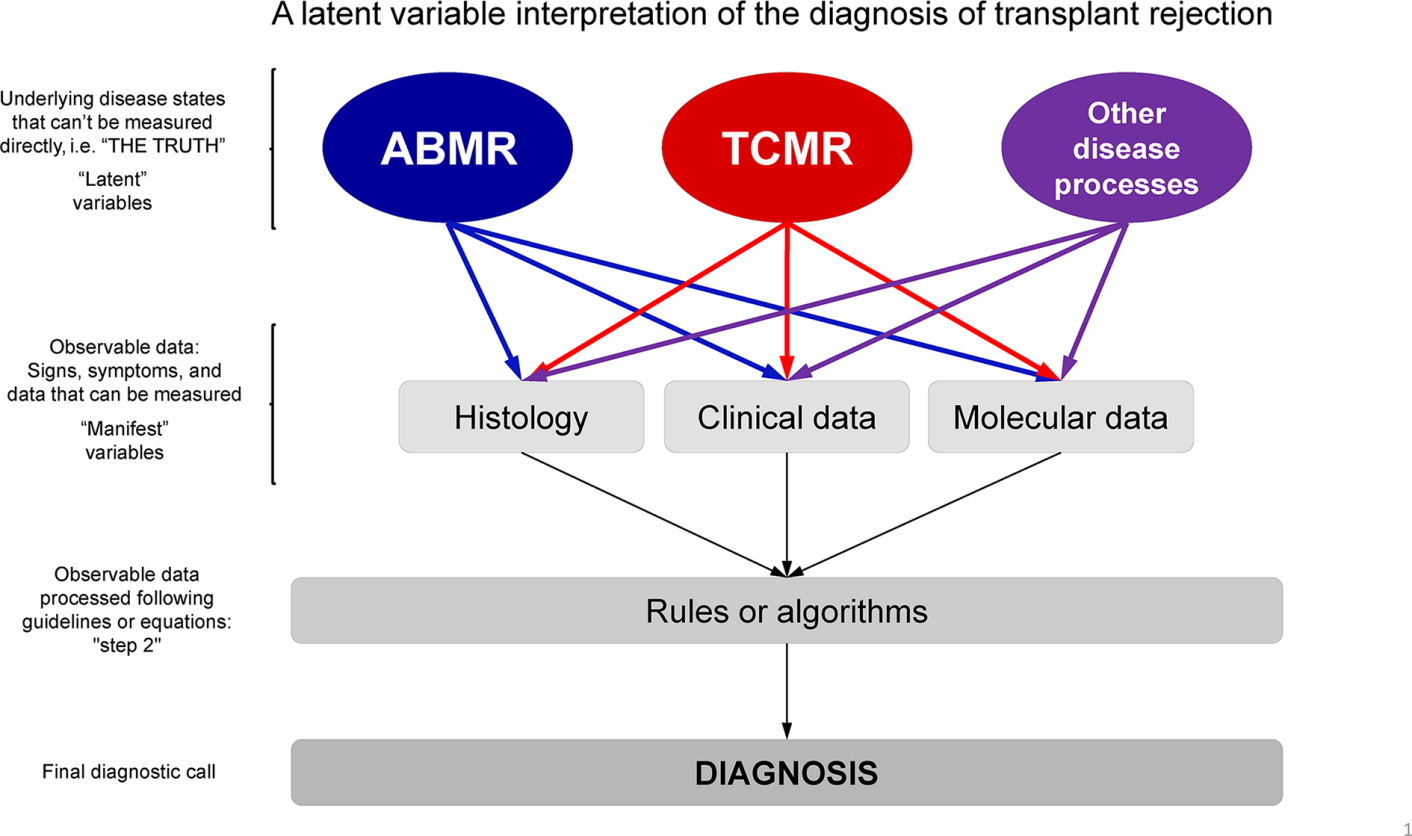
Latent variable interpretation of transplant rejection True disease states are ‘latent variables’, that can seldom be known with absolute certainty. Observable measurements (‘manifestations’: histologic, clinical, and molecular data) of the underlying diseases are used to assign a diagnosis. The Banff system uses histologic lesions + DSA + C4d (step 1) to make diagnoses using consensus rules/expert opinion (step 2). MMDx measures gene expression (step 1) to assign disease states/probabilities (step 2) using: (**A**) Scores from supervised methods – classifiers based on correlations/associations between gene expression and histologic diagnoses/lesion scores. (**B**) Unsupervised methods combining scores from (A) and gene set (PBT) scores. Once in place, both Banff and MMDx require only one type of data to assign diagnoses in new samples – histology/DSA for Banff, and gene expression for MMDx [1].

The concept of ‘deep medicine’ and use of artificial intelligence, statistics, and machine learning has gained prominence in medical diagnostics over recent years [45]. The ability to predict outcomes, provide probabilities, and offer objective diagnoses is especially relevant in areas of medicine where the standard of care is less reliable [45]. If properly developed, i.e., with regards to minimizing over- or underfitting, machine learning algorithms can provide high quality, robust, and precise diagnoses [10]. In some situations, results produced from machine learning algorithms are also *more likely* to be correct with regards to the true disease phenotype than their standard of care counterparts (e.g., histology and clinical data) that rely on visual assessments or consensus-driven diagnostic guidelines [10]. It is important to remember that the truth in diagnostics is always unknown – a latent variable (Figure 1). All diagnostic systems are attempting to estimate the true disease state in the patient. Each system employs certain observable metrics (step 1) interpreted through rules or algorithms (step 2) to make this estimation – Banff guidelines use lesion scores as assigned by microscopic examination and the overall combination of scores leads to a diagnosis, while molecular systems like MMDx use precise objective measurements (in MMDx, gene expression) interpreted by ensembles of machine-learning algorithms. These will be interpreted by the clinician in the context of other clinical tests. While algorithms cannot replace clinicians, the objective data produced by machine learning can increase the quality of (and confidence in) the final diagnoses [45].

MMDx machine learning analyses can take two forms: supervised i.e. by training on conventional disease labels; or unsupervised, i.e., based solely on the structure of the molecular data. The overall goal is to make accurate assessments of rejection and injury and understand the risk of progression to failure.

### Supervised analyses with microarray data

Supervised analyses use sample labels to train an algorithm. In this way, numerical data (e.g., transcript expression from a set of biopsies) is combined with and informed by its labels (i.e., clinical, histologic, or biochemistry data in these analyses). Specific patterns or results in the expression data that correspond to a particular feature can be detected and their predictive potential for that feature evaluated.

MMDx has incorporated many types of supervised analyses into the final ensemble of algorithms – the primary examples being trained classifiers. However, training an algorithm with supervision by a particular label does not necessarily mean that the resulting algorithm is predicting that label in an unknown sample going forward – for example, this does not imply that a classifier trained on DSA will be used to assess DSA in new samples. Since the DSA testing results are available by other means, this would not be as useful. Instead, the classifier is *predicting the presence or absence of DSA-associated abnormalities in gene expression*, and in the case of the DSA_Prob_ classifier (classifier trained on DSA-positive vs. DSA-negative labels [46]), provides an estimate of molecular ABMR.

**Figure 2:**
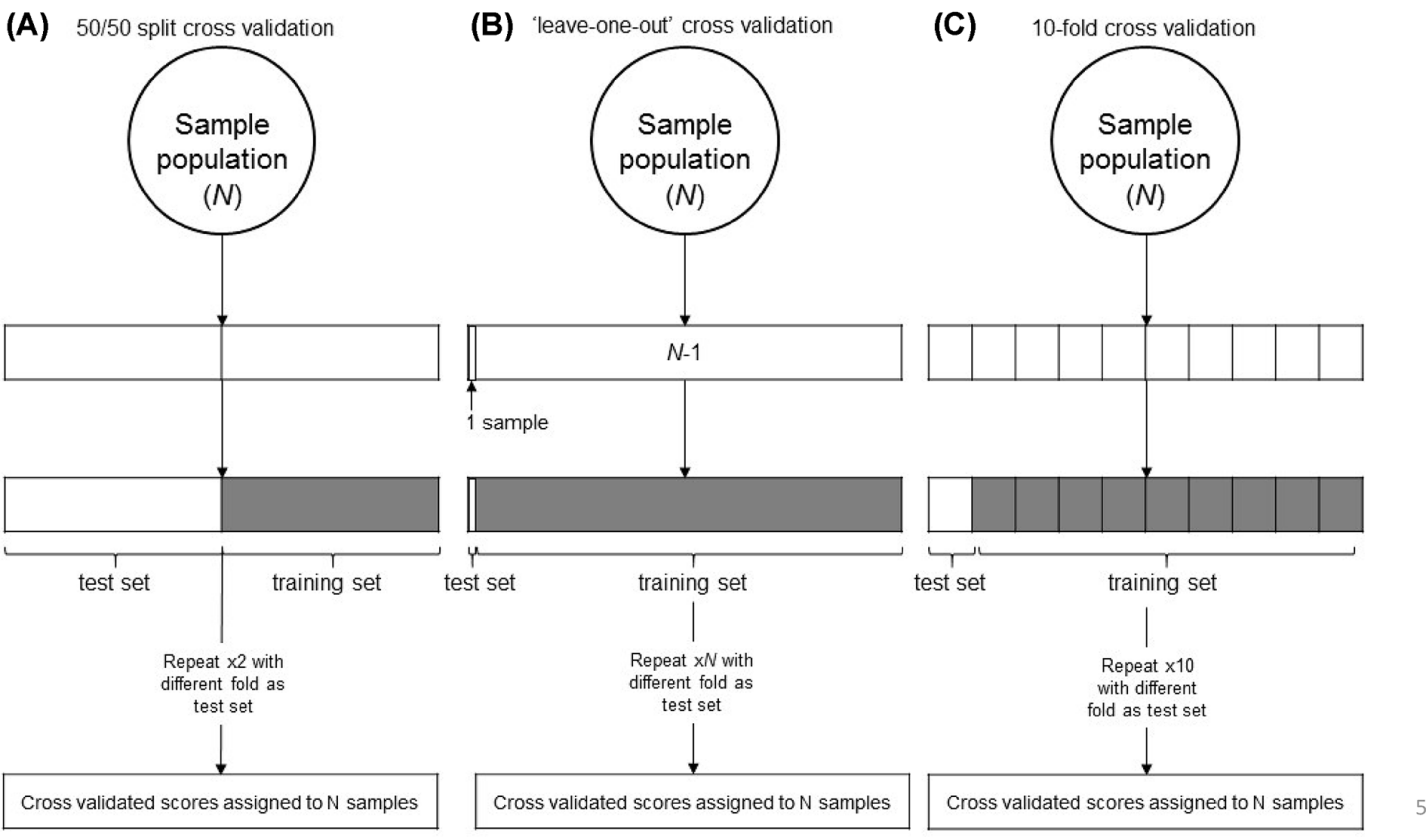
Various cross-validation methods used to assign machine learning scores to a sample population A sample population with size *N* is split into a training set and test set (size varies with the methodology chosen). The algorithm is trained in one set and tested in the other to avoid training and testing in the same population (overfitting). The end product is a population where every sample has an assigned score, and no sample score was assigned by an algorithm that was trained on that particular sample. (**A**) 50/50 split cross-validation (we note that in practice, this method involves training in half the population and testing in the other half, but the reverse training/testing combination is not done. If the reverse is not done, only half the samples get tested as well as only half of them getting trained.) (**B**) ‘Leave-one-out’ cross-validation, where the test set comprises only one sample, and the algorithm is trained in the rest of the population (*N* − 1). (**C**) Ten-fold cross validation, where the sample population is split into tenths, one tenth is used for testing and the remaining nine tenths used for training – the entire process repeated ten times so that every sample is used for both training and testing, but scores assigned to each sample are from an iteration where they were not included in the training set [1.

To generate a machine learning algorithm (e.g., a classifier), a data set of samples plus their accompanying standard of care information is divided into two groups for cross-validation. The goal is to establish how well the algorithm will perform on future unknown samples. Groups can be equal (e.g., a 50/50 split of the data set for training and testing, respectively, Figure 2A) or unequal (e.g., train on the entire set minus one, test in the one sample as done in ‘leave one out’ methods, Figure 2B). Splitting groups 50/50 offers the advantage of testing and training in an evenly-sized set of samples but does restrict the samples available for training, while training in a larger set of samples (for example, ‘leave one out’) offers the advantage of giving the algorithm more initial information on which to base its predictions but also reduces the size of the test set. When estimating the performance of an MMDx classifier, we use 10-fold cross-validation (Figure 2C) – i.e., splitting the population into tenths, then using nine-tenths as a training set and testing in the left-out one-tenth. This is repeated 10 times until all samples have been assigned a score in an instance where they were not part of the training set. Ten-fold cross-validation allows us to maximize the information available from the dataset while minimizing overfitting – a large number of samples are used for training, but samples are never assigned a score in the test set from an instance where they were also used in the training set.

Ideal algorithms will (1) be able to assign accurate values within the existing data set, showing that it fits the current data closely, and (2) be able to assign highly accurate results in a *new* data set in which it was not trained. However, these two principles are in conflict (bias-variance tradeoff) [47]. An algorithm should avoid fitting the current data set too closely within the population used for training (overfit), especially if the problem is more complex, or it may underperform and misrepresent future subsets with a different case mix. Likewise, an algorithm must not be so loosely fit to the current data that it has not ‘learned’ the appropriate information to make the designated prediction in the current or future dataset (underfit). The bias and variance must be balanced in order to produce an algorithm that performs well in the current data and is usable in new, unknown populations.

Machine learning algorithms are diverse (e.g., linear discriminant analysis and random forests), and each method approaches a problem from a different perspective. Several algorithms can be combined by taking the mean or median value of all estimates (called a consensus or ensemble approach) [10,48–53]. Statistical and machine learning literature discuss the increases in accuracy and stability when consensus approaches are used; the use of an average of multiple ‘observers’ or algorithms is more likely to be correct than any single observer [54].

**Figure 3:**
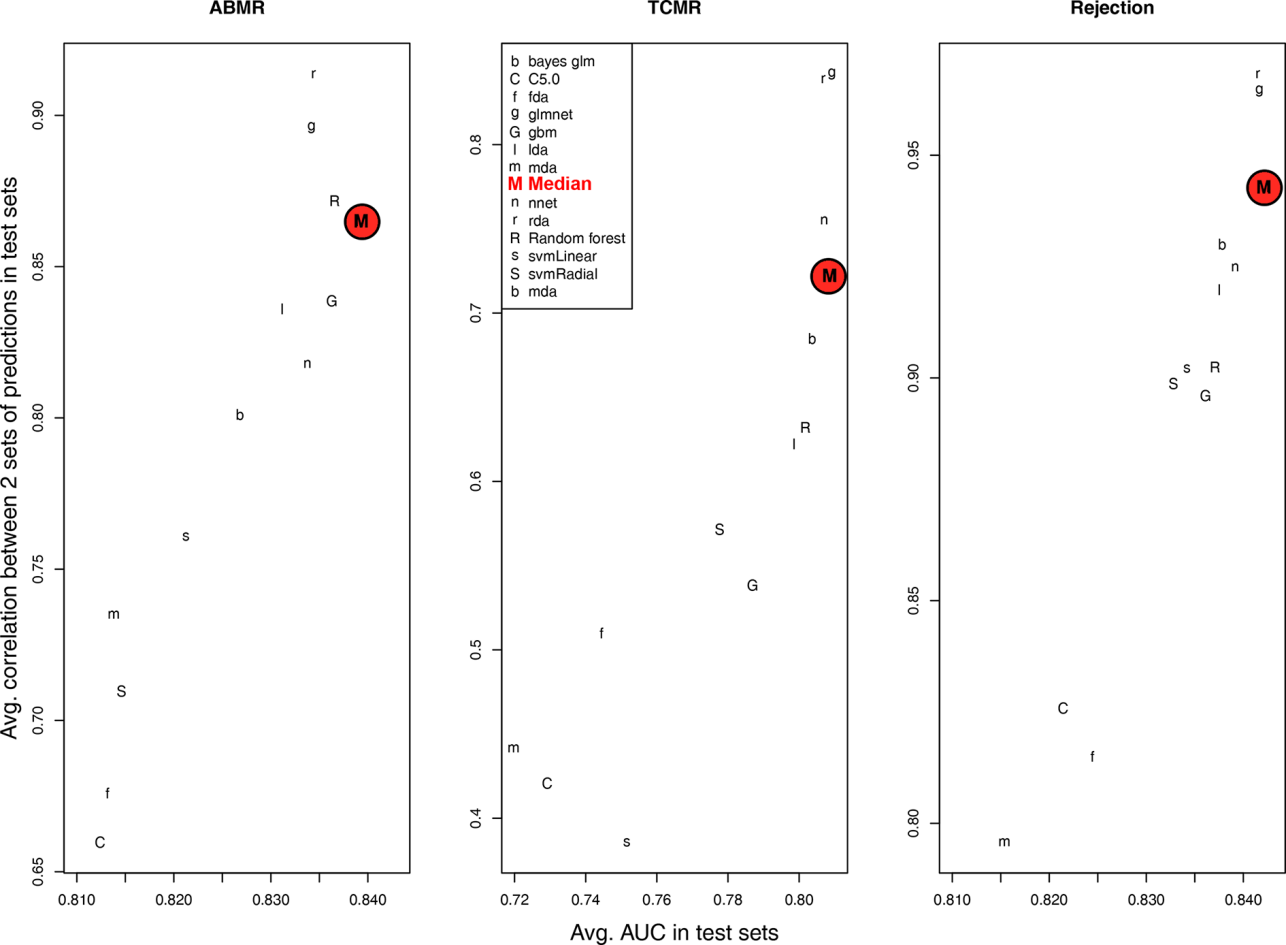
Stability vs AUC (area under the receiver operating characteristic curve) for predicting histology diagnoses Visualization of each algorithm performance plus the median (M) based on resampling the combined *N* = 1679 population in (**A**) ABMR, (**B**) TCMR, and (**C**) All rejection. Stability is assessed as the correlation between test set predictions from two separate training sets. The median (‘M’, here highlighted in red) has a higher AUC than all the individual tests for ABMR, and the second highest for TCMR (for details of the type and *N* of resampling, see [10]).

In cases where a single machine learning method is used, there is typically no *a priori* method of determining which algorithm will be best for the data set or the question asked (the ‘No Free Lunch’ theorem) [55]. Instead, the choice of machine learning algorithm is conventionally based on practicality (e.g., ease of use, availability of software, etc.). No machine learning method will perform better than all other methods in all possible datasets, as demonstrated in the varying AUCs for predicting ABMR (Figure 3A), TCMR (Figure 3B), and all rejection (Figure 3C) for each of the 12 machine learning algorithms used in ABMRProb, TCMRProb, and RejectionProb MMDx classifiers, respectively (classifiers trained on the standard of care labels for ABMR, TCMR, and all rejection [10,36,56,57]).

**Figure 4:**
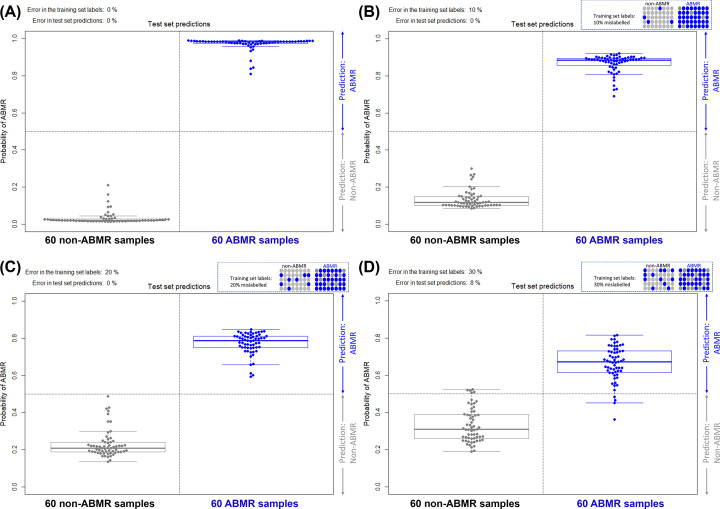
Test set predictions when error is intentionally introduced through label flipping Test set predictions separating ABMR samples from non-ABMR samples are shown when the training set contains (**A**) 0%, (**B**) 10%, (**C**) 20%, and (**D**) 30% error introduced intentionally by inverting labels prior to training. In each case, the true ABMR and non-ABMR labels applied by the algorithm separate, with the error in the test set predictions substantially less than that introduced in the training set.

**Figure 5:**
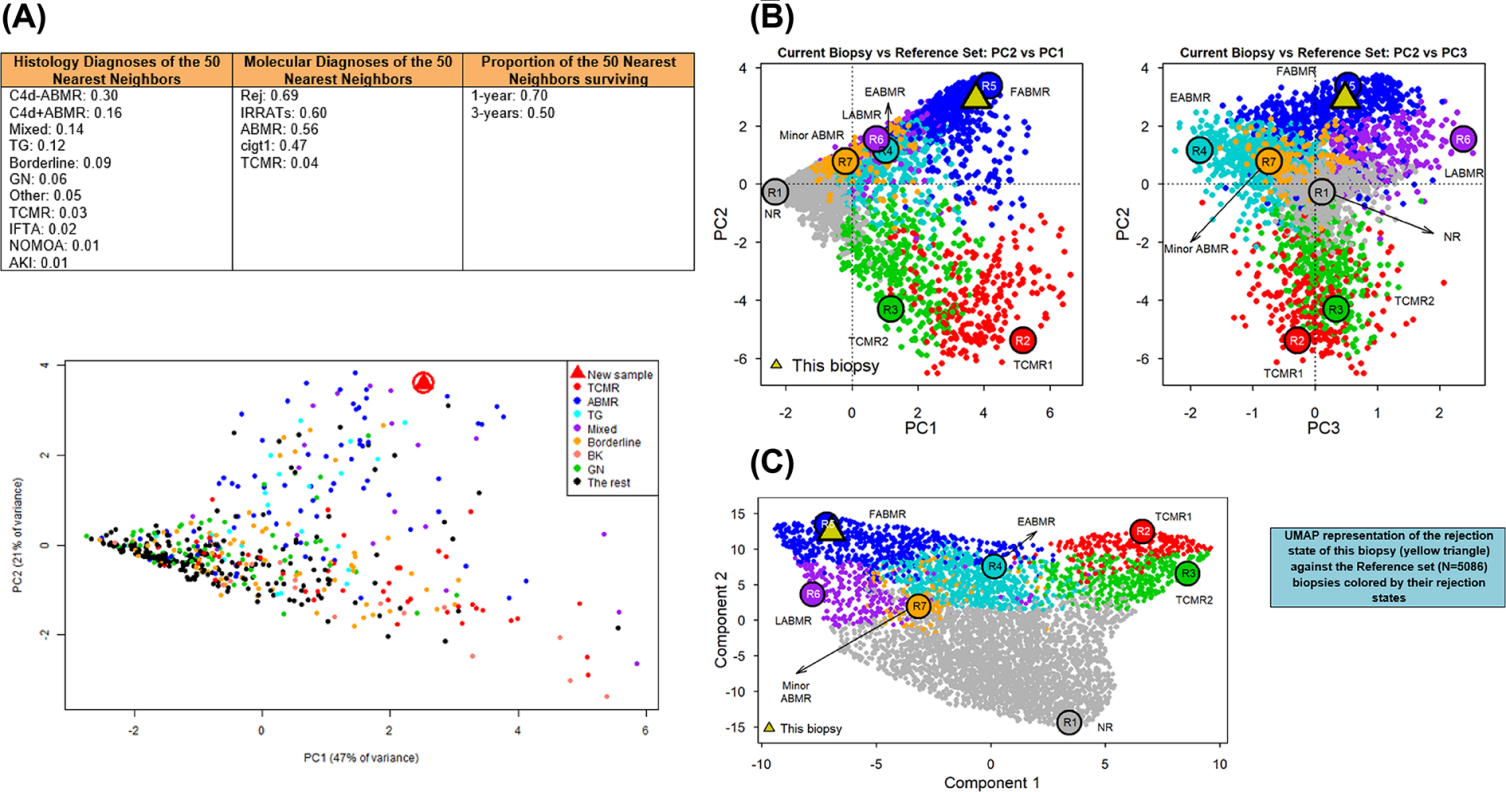
Various visual outputs used by MMDx on reports throughout the INTERCOMEX study (**A**) The nearest neighbor visualization approach used on early MMDx-Kidney reports, using the relevance of biopsy-to-biopsy distance in principal component analysis to draw conclusions about an unknown biopsy based on its proximity to known ‘neighbors’ in the figure. (**B**) More recent visualization of an unknown biopsy on the MMDx-Kidney report, removing a formal ‘nearest neighbors’ comparison, but showing the biopsy against the known population in PC1, PC2, and PC3. Finally, (**C**) a UMAP representation of the MMDx-Kidney biopsy population, where the no rejection biopsies are visually resolved rather than tightly grouped.

The MMDx machine learning algorithms are able to correct a certain amount of error in the training set used for supervised analysis [10] (Figure 4). In a series of tests where a certain amount of intentional error was introduced into the classifier training sets for a well-established label (here we use the ‘ABMR’ diagnosis as an example, defining intentional error as a sample identified as ‘ABMR’ per clinical standard at the local center, but entered as ‘Not ABMR’ into the dataset for the purposes of this analysis, and vice versa), we found that the classifier was able to correctly reassign many of those labels in the test set using only the gene expression data. Test set predictions separating ABMR samples from non-ABMR samples are shown when the training set contains 0% (Figure 4A), 10% (Figure 4B), 20% (Figure 4C), and 30% error (Figure 5D), introduced intentionally by inverting labels prior to training as described. In each case, the true ABMR and non-ABMR labels applied by the algorithm separate, with the error in the test set predictions substantially less than that introduced in the training set. In fact, errors present in the test set predictions remained at 0% when up to 20% error was introduced, and was only 8% when 30% error was introduced. While the effect is shown here using ABMR as an example (a phenotype with moderate to high correlation with gene expression), the ability of the classifier to overcome labeling errors would be reduced in more subtle disease processes, i.e., where the correlation between the true phenotype and gene expression is weak or highly variable. The combination of phenotype label quality and strength of molecular associations will determine the degree to which a classifier can correct the mislabeling.

Classifiers are often criticized based on the quality of the labels used for training – under the assumption that if the labels are largely unreliable, the classifier will be unreliable to the same extent. However, these findings show that error (up to a certain limit) in the training set can be overcome to an extent by the classifier, as it bases its calls on the typical molecular characteristics of the training set cases. This process means that the classifier can handle a certain level of ‘noise’, or incorrect labels, as long as the signal in most of the biopsies is relatively consistent. Figure 4 also illustrates how overfitting in a given population can be unwise – it can lead to training on incorrect labels more prominent in the initial population. This figure presents an example with simplified conditions – in reality the extent to which noise can be overcome depends not only on the fraction of noise within the labels but also on the *N* of the training and testing population, and the strength of the correlation between the ‘true’ underlying phenotype and the gene expression values. Additionally, the true underlying phenotype is continuous rather than binary, therefore the classifier will also depend on the distribution of the training samples along that disease spectrum. Nevertheless, it is an important to conceptually understand that the performance of a classifier is not directly or proportionally affected by the errors present in the labels as assigned in the training set, but rather has a more complex relationship with the initial data.

### Unsupervised or semi-supervised analysis with microarray data

Unsupervised analysis does not use labels to inform the data, e.g., uses only molecular data with no involvement of standard of care categorizations. An unsupervised analysis of a given population can be achieved using clustering methods (i.e., archetypal analysis or k-means clustering). Samples are grouped based on similarities found in the data, which may or may not correspond to groups assigned by other labels. Some forms of unsupervised analysis provide only group-based information, failing to recognize that some biopsies are closer to group boundaries than others (e.g., standard k-means). Other forms recognize the relationship between samples and assigned groups while still assigning distinct clusters (e.g., archetypal analysis and fuzzy k-means clustering). An advantage of unsupervised analysis is especially apparent in data sets where the gold standard class labels are unreliable, and the signal-to-noise ratio is too low for reliable supervised analysis. Another benefit is the unbiased nature of unsupervised methods – where the approach uses the raw data only and is minimally or not affected by opinions or subjectivity that may accompany label assignment.

MMDx uses archetypal analysis over other clustering methods [7,12,58]. Archetypal analysis is a clustering method that identifies a selected number (*n*) of theoretical idealized extreme phenotypes called archetypes (e.g., A1, A2, A3, etc.), and assigns each biopsy *n* scores to describe its proximity to each archetype (e.g., A1score, A2score, A3score, etc.) [12]. By convention, archetypal analysis assigns biopsies to clusters based on their highest archetype score, objectively classifying biopsies for analysis. Typically, the scree plot ‘elbow method’ plus knowledge of clinical phenotypes and previous experience [12,15,59] determines the appropriate number of groups in an analysis. With these characteristics, archetypal analysis allows for the assignment of clusters, while also preserving the uniqueness of each biopsy and heterogeneity of the population through the archetype scores. Archetypal groups are useful as an objective classification of the biopsy population based on the most dominant sources of variation in the data, while the archetype scores give continuous numbers useful for studying relationships with the clinical, histologic, and molecular phenotypes.

## Visualization strategies – clinically relevant representations of microarray data

Data sets can be visualized in several ways that each afford unique advantages and disadvantages to the observer. MMDx uses two main visualization methods to report biopsy findings and study populations: Principal Component Analysis (PCA) and Uniform Manifold Approximation and Projection (UMAP). These methods differ in many ways, including (1) the relevance of distance between points (biopsies) on the plot, and (2) if points (biopsies) are permitted to be grouped tightly, i.e., overlaid on the plot in a small space, as in PCA; or if the points must be spread out so that each biopsy can be visually resolved, as in UMAP.

PCA uses a set of designated inputs to distribute the data based on those features (i.e., using graft rejection-associated features to distribute a set of biopsies based on their rejection-ness). PCA presents the advantage of meaningful distance between samples – if two samples are grouped closer based on their PC scores, it indicates a higher degree of similarity between those samples. This is particularly useful in medical diagnostic applications, where a new biopsy can be projected against a PCA plot of samples with known phenotypes or outcomes, and therefore that new biopsy can be compared with those phenotypes or outcomes using its location on the plot. This benefit has been employed on MMDx reports, both in past reports to show the outcomes of the ‘nearest neighbors’ (Figure 5A), and in the context of showing a new sample overlaid on a known population (Figure 5B, same biopsy shown across plots) [11]. However, this advantage can also obscure data – if a large group of biopsies are very similar in terms of the features used in the PCA, they may be grouped very tightly and ’stacked’ over each other, making them difficult to individually visually resolve and possibly obscuring the number of biopsies present in that plot area. PCA has an infinite number of available dimensions to describe variation in the population – for MMDx purposes, we typically limit the dimensions to three (PC1, PC2, and PC3) and find that this describes most of the relevant variation in the population while also containing enough variation per component to allow us to assign meaning to each component. For example, in kidneys, variation across PC1 is ‘good’ vs. ‘bad’ or ‘no rejection or injury’ vs. ‘any rejection or injury’, while variation across PC2 is ABMR vs. TCMR, and across PC3 is stages in ABMR – early-stage, fully developed, and late-stage [12].

UMAP is an alternative visualization method that can compress all variation into two dimensions if required: Component 1 and Component 2, allowing variation to be displayed on a single plot. On MMDx reports, UMAP uses the exact same input data as the PCA. UMAP also differs from PCA in that all samples are distinct on the plot and not ‘stacked’, making them easy to visually resolve from each other, but as a result distance is no longer meaningful for quantitative analysis. UMAP has been developed to ensure that similar samples are close together (it preserves local similarities), while being largely unconcerned with the relative placement of more distantly related samples. Current MMDx reports have incorporated UMAP plots, to better show the No rejection biopsies and other groups that tend to cluster tightly together in a small area in PCA (Figure 5C). Since recent MMDx studies have shown more rejection activity than originally recognized within biopsies called No rejection by MMDx, histology, or both – visually resolving the entire population of No rejection biopsies becomes even more important [46,60].

For the purposes of MMDx, we use both PCA and UMAP. PCA allows us to use what we know about a biopsy population to interpret a new biopsy, to see how groups of biopsies can be more or less alike, and PCA scores can also be used for downstream analysis at a later time. UMAP allows us to easily resolve individual biopsies, even when they are in a comparatively more homogenous group, i.e., ‘no rejection’.

## Microarrays as a platform versus alternative technologies for studying gene expression – choosing a platform based on specific research questions

In developing a molecular diagnostic system, the details of the platform and reagents to be used are critical. The security of supply and the key reagents over time, and the commercial viability of the supplier, must be paramount to avoid being orphaned in the future if a critical element can no longer be obtained. Knowledge of the biologic process is one thing, but switching between platforms can be a significant and costly issue, since it means that the entire system must be redeveloped, for example, the machine learning algorithms. Even two supposedly identical machines can give different measurements, and must be constantly recalibrated if the standardized machine learning algorithms are to be applied to the measurements coming from different machines.

### Formalin-fixed paraffin embedded samples

The emergence of molecular assessment of biopsies has underscored the limitations of conventional methods and created pressure to bring molecular diagnostic methods into the clinic [11,35]. The diagnostic use of gene expression data optimally requires the assessment of many transcripts, interpreted using algorithms, for example, classifiers [12], with rigorous standardization of the measurements. Gene expression studies usually rely on high-quality RNA extracted from tissue that has been either snap*-*frozen or stabilized in a medium such as RNA*later*™ to prevent RNA degradation [61]. However, because formalin-fixed paraffin-embedded (FFPE) tissue is the standard for histologic diagnosis, there is interest in using FFPE biopsies for studies of gene expression, particularly when RNA*later*™-stabilized or frozen specimens are not readily available. Working with RNA from FFPE specimens presents significant challenges, including variability in tissue handling/processing, length of storage, and RNA extraction methods that affect the quality of RNA [62]. The most significant challenge is that formalin causes RNA–protein and nucleic acid cross-linking, and chemically modifies RNA by adding mono-methylol groups to 40% of adenines and 4% of uracils [63]. RNA extracted from FFPE tissue has low yields, and the purified RNA is shorter, more degraded, and without poly-A tails, requiring a suitable reverse transcription protocol for expression profiling [64]. The smaller fragments of RNA are less efficient templates for hybridization, reverse transcription, or amplification [62], even using newer modifications [65].

### RNA sequencing

RNA sequencing is an outstanding research tool with many advantages, and is used in a large variety of ways differing in depth and technical details. While RNA sequencing is the newer technology, we choose microarrays because they are thoroughly validated, well-established, well-documented, and readily available – all advantageous features for a commercial platform and real-time analyses. The information created from a single sample is less cumbersome to store, but still extensive enough to provide many opportunities for post-hoc analysis. Results are available within 1–2 days; all sample types are valid for analysis, cost is relatively low per sample, no batching is required, and arrays are suitable for large scale studies (e.g., thousands of samples). RNA-sequencing does allow for better coverage of genes, especially those with low expression, so in some contexts can be an important tool. However, deep sequencing is not always needed, and can produce extraneous data unnecessary for the research question. For these reasons, we continue to use genome-wide microarrays for MMDx testing, as this depth of data is sufficient to answer most or all of our research questions.

### NanoString nCounter

Microarrays are also often compared with gene expression as measured by the NanoString nCounter system. The nCounter system is an adaptation of the microarray method, but these methods differ in several ways – one of the most important differences is that it must use pre-selected gene lists and thus cannot be used as a genome-wide discovery platform. Similar to the questions that must be asked when considering RNA sequencing, one must consider what information they want to obtain prior to choosing between nCounter and microarrays. If discovery is an important element of the study (as in previously published MMDx analyses), NanoString should not be used. While NanoString nCounter does provide some advantages, i.e., FFPE material is stable for long periods of time, readily available, and can be easily sourced, it is not the appropriate platform for all research applications.

### Considerations when choosing a platform

Choice of platform [65–70] remains an important element of any study, or the development of any commercial product. Prior to choosing a particular platform, some critical questions should be asked (Table 1). Time-to-results of a test is a significant consideration if a discovery platform will also be used for commercialization of a test – batch dependency can create delays unless a test is high throughput. Reproducibility between test runs ensures that past, present, and future test results can be compared for a single patient, while minimizing data drift in the entire test population. The platform chosen should be appropriate based on the specific biological question being asked by the test, providing the information necessary to resolve that question as well as potentially relevant post-hoc analyses without overloading the test with too much extraneous data. If the test may be commercialized in future, cost per test should be considered as extremely expensive tests may limit real-world availability. The number of samples processed by the test in a single run should also influence platform choice – some platforms are flexible and allow for a single sample or multiple to be run, but others require several samples per run making them more restrictive. Will the platform be needed for discovery research, or simply to measure expression in genes that are already identified? Finally, the central or local nature of the test must be considered: if a test is to be run locally, the platform must be ubiquitously available and easily standardized to ensure precision of results, and if a test will be centrally run it must support processing of samples in formats that ensure stable shipping from multiple regions.

**Table 1:** Questions to consider when choosing a technology for analyses

#	Question	Description
1	Will test results be needed ‘in real time’ or is batching acceptable?	This aspect should be considered as it affects delivery time of results.
2	Is reproducibility between test runs important?	Tests differ in documented reproducibility, this becomes important if samples are small or may be run more than once.
3	What biological question is being asked?	For example: are you looking for disease vs. no disease? Disease vs. disease? Is sensitivity more important than specificity, or vice versa?
4	Will the test be commercialized, and should per test cost be considered?	If for research purposes only, cost per test is only a limitation of research budget. If eventual commercialization, insurance approval etc should be considered.
5	What information do you need from the test?	Do you need only a few measurements? Do you need deep sequencing? Can your analysis be achieved using a limited set of probes?
6	How much information from the test do you intend to store?	Do you have the capability to store TBs or only GBs of research data from your analyses?
7	How many samples will you be processing at one time?	Will you be running 1–10 at a time, or 30+ at a time?
8	Will this be a central or locally run test (using kits)?	Centrally-run tests have more flexibility, but locally run tests must consider how prevalent the equipment is across centers, how much it costs, and how well it can be standardized. Tests run locally using kits tend to give variable results. This can be a significant problem if machine learning classifiers are to be used.

Throughout the development of MMDx, we have chosen microarrays for a series of reasons (Table 2). Affymetrix microarrays have controlled costs associated with them – the process is identical from sample to sample (e.g., reagents, style of microarray chip, equipment used, labor time required, etc.). The genome-wide coverage gives enough data for all required analyses, and enough possible probes to allow many different analyses from the same datasets without the data size being cumbersome or challenging to store (e.g., gigabytes versus terabytes of data storage requirements). Microarrays are a mature technology and thoroughly documented for manufacturing standards [71] including all associated materials, reagents, and machines, allowing the research questions instead to focus on what is seen in the data itself rather than how to interpret the style of data. Manufacturers provide ongoing security in availability, avoiding the risk of a technology becoming orphaned or abandoned after it's put into commercial use. Microarray processing allows for the analysis of single biopsies or batches depending on what is needed at the time, so results can be provided in real time without needing to wait for a batch collection. The same Affymetrix technology can be employed in multiple laboratories internationally and standardized, allowing for machine-learning results to be shared between centers. Extracted biopsy mRNA can be stored long-term for future analyses on alternate platforms, or for repeated sample runs, providing a stable library of past samples. Finally, a consensus has been available for the best practices on analyses (including approaches, methodologies, validation steps, etc. [72]) for many years, removing unnecessary guesswork and increasing ease of use.

**Table 2:** Reasons for the choice of Affymetrix microarrays

1.	Controlled costs
2.	Genome-wide coverage with excellent reproducibility of measurements and internal controls
3.	Mature technology (old) with high manufacturing standards of all materials and reagents and machines
4.	Ongoing security of supply of all reagents from providers that are commercially viable long-term
5.	Ability to process single biopsies, and not rely on batches assures no batch variation
6.	Ability to use the same technology in service laboratories as in the discovery center facilitates updates and standardization across different sites
7.	Ability to retain biopsy mRNA for future analyses on other platform
8.	Consensus regarding best practices for analysis (approaches, methodologies, validation, etc.) has existed for ∼15 years

## Standard of care local histology versus central histology

The clinical trials that led to the MMDx system compared the MMDx results with standard of care local histology assessments. While central review of histology eliminates a source of variance by nature of being assigned by a single observer (increased precision), it is not the way diagnoses are actually made in practice. Management decisions are made based on locally-assigned histology, clinical features, and the clinician's knowledge of the patient. Our goal was to understand how MMDx related to the current standard of care.

Any metrics assigned by a human observer, regardless of how neutral they may be, will contain some level of bias based on how they interpret the diagnostic rules, especially if those rules contain any ambiguity or subjectivity. A panel of experienced pathologists, attempting to use the ‘median’ opinion and avoid outliers, is probably more accurate. We would like to have a comparison between MMDx and the opinions of a central panel of experts but this is a different question. Additionally, having multiple central reviewers examine the same set of biopsies and assign histologic feature scores is expensive and would then need to be compared with the local standard of care assessments, and central reviewers will not always agree with each other [56].

The triple comparison between local standard of care, MMDx, and central review would be a useful target for a future study. Meanwhile, MMDx has chosen to train on the standard of care histologic features in INTERCOMEX (ClinicalTrials.gov #NCT01299168), INTERHEART (ClinicalTrials.gov #NCT02670408), INTERLUNG (ClinicalTrials.gov #NCT02812290), and INTERLIVER (ClinicalTrials.gov #NCT03193151), and the Trifecta studies (Trifecta-Kidney ClinicalTrials.gov #NCT04239703, Trifecta-Heart ClinicalTrials.gov #NCT04707872, Trifecta-Lung ClinicalTrials.gov #NCT05837663).

## Translating principles in one transplanted organ population to others – gene expression can be ‘organ-agnostic’

An important finding throughout the MMDx projects has been that many gene expression patterns are related to disease processes in general tissue, rather than to disease processes in specific organs [1,2,73]. MMDx studies began in kidney transplants, training supervised algorithms on Banff features (e.g., g-lesions, ptc-lesions, and DSA status) and diagnoses (e.g., ABMR and TCMR), and developed unsupervised or semi-supervised phenotype classifications to describe the population (e.g., archetypal clusters and PC scores).

### Molecular scores used to translate features of molecular rejection and injury between organs

**Figure 6:**
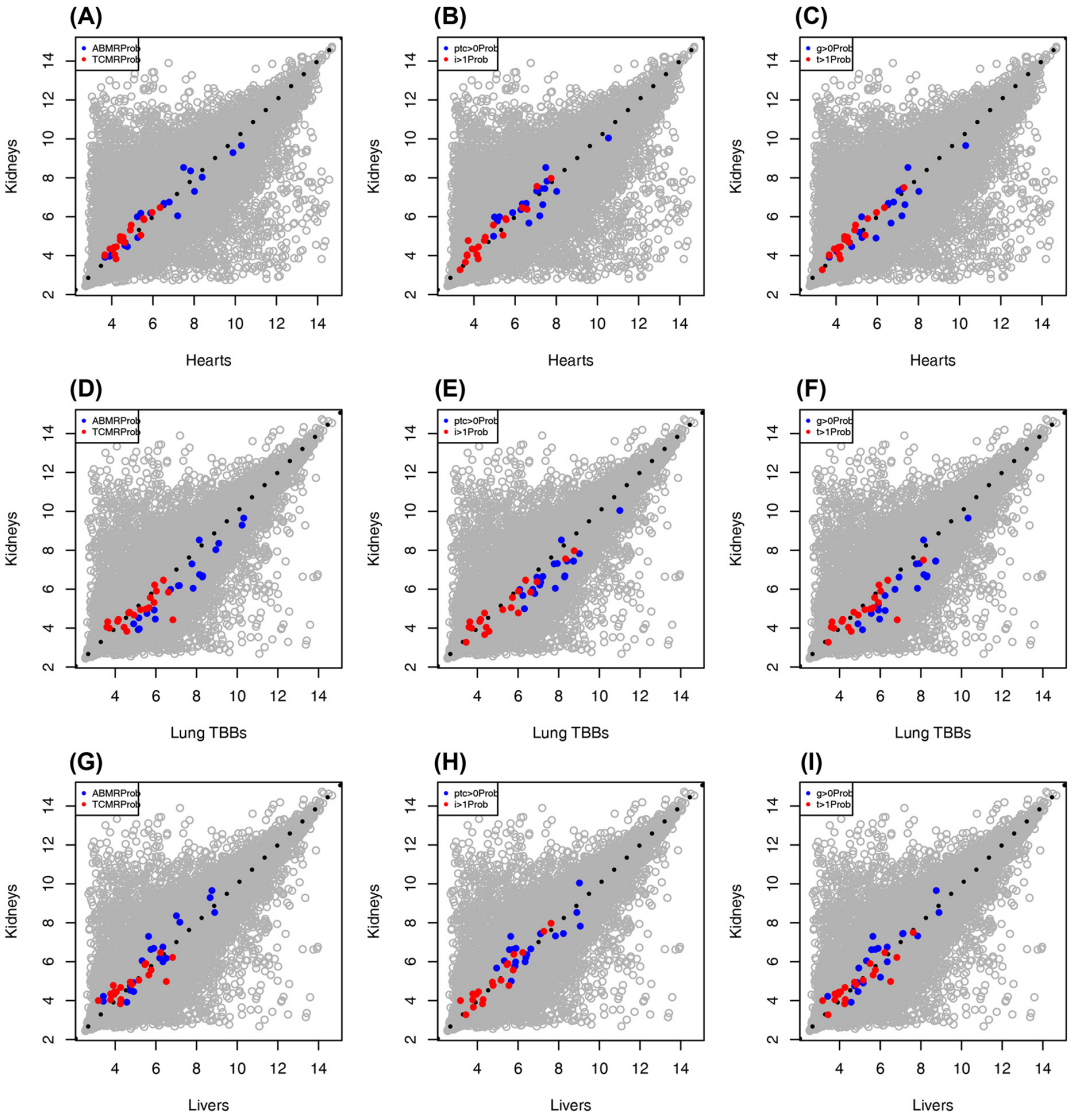
Cross-organ comparisons showing molecular classifier scores in transplanted kidneys versus hearts, lungs, and livers Classifier scores have performed reliably in all MMDx studies, reflecting considerable sharing of biological mechanisms across organs, and allowing us to study trends between various organ populations. Each dot represents a particular gene assessed by the microarray chip, and the top 20 genes used in each classifier are colored (ABMR-associated features in blue, TCMR-associated features in red). (**A–C**) ABMR-associated classifiers (ABMR_Prob_, ptc>0_Prob_, and g>0_Prob_) and TCMR-associated classifiers (TCMR_Prob_, t>1_Prob_, and i>1_Prob_) top transcripts shown in transplanted kidneys (*y*-axis) versus hearts (*x*-axis). (**D–F**) shows the same analysis but in transplanted kidneys (*y*-axis) versus lungs (transbronchial biopsies, *x*-axis). Panels (**G–I**) show the same analysis, but in transplanted kidneys (*y*-axis) versus livers (*x*-axis).

Kidney transplant-derived transcript sets and even classifiers like the ABMR_Prob_ classifier [10,57] have been used in assessing other organ transplants biopsies where the training of such classifiers is limited by extensive interobserver disagreement in diagnoses. The kidney transplant core biopsy assessment using Banff guidelines offers more detail because core biopsies are more easily read and the lesions are scored individually as well as used in assigning diagnoses. As heart and lung biopsies are much more difficult to interpret histologically, we use algorithms trained on kidney histology to interpret molecular states on the assumption that some of these molecules are shared across organs in the same state [74]. The high conservation of gene expression permits transcript sets initially developed in mouse models or cell lines to be used in kidney biopsy interpretation, and by extension in other organ transplant populations, including hearts, lungs, and livers. These classifiers were originally developed in kidney transplant populations using Banff lesion scores, standard of care biopsy features (like DSA status, GFR, and proteinuria), and final Banff-based diagnoses to train or ‘supervise’ the classifiers [10]. These clinical and histologic features were considerably more reliable and defined than their counterparts in heart, liver, or lung transplantation, where the diagnostic systems are increasingly subjective and kappa values are lower between observers [37,40,75–83]. We found that moving these classifiers between organs allowed for the study of various biopsy features regardless of population, and that probes used for these classifiers performed similarly between organ populations (Figure 6, ABMR-associated features in blue, TCMR-associated features in red). While a classifier may be trained on ABMR or TCMR diagnoses in kidney transplantation (ABMR_Prob_ and TCMR_Prob_, respectively), that same classifier was able to detect ABMR-like features in heart transplants (Figure 6A–C), lung transplants (Figure 6D–F), and liver transplants (Figure 6G–I). Classifiers may be trained on a particular feature, but use organ-agnostic molecular expression associated with the presence or absence of that feature for predicting disease, injury, or rejection in a new biopsy. This allowed for classifiers developed in kidneys to also detect injury, loss of function, and rejection in other organs.

Experience with the pathogenesis-based transcript set scores (e.g., injury and repair-associated transcripts ‘IRRATs’) was the original basis for this concept of translational ability across organs. PBTs were developed and annotated in previous analyses in human cell lines, mouse experimental models, and human transplant biopsies [84], and are associated with biological mechanisms in rejection and injury (https://www.ualberta.ca/medicine/institutes-centres-groups/atagc/research/gene-lists). Ultimately, these findings show that the molecular processes operating in rejection episodes, or when parenchymal tissue is injured, can be similar regardless of which organ they occur in.

PBT values represent the mean fold difference in expression values of that set of transcripts in a population of biopsies compared with a selected control group. Control groups vary according to the organ and the analysis. PBTs (Table 3) [1] are defined as follows: ABMR-associated transcripts (ABMR-RATs) [85], alternative macrophage-associated transcripts (AMATs) [86], B cell-associated transcripts (BATs) [87], cardiac injury and repair-induced transcripts (cIRITs) [88], damage-associated molecular pattern-associated transcripts (DAMPs) [89], endothelial DSA-selective transcripts (eDSASTs) [90], endothelial cell-associated transcripts (ENDATs) [91], γ-interferon and rejection associated transcripts (GRITs) [92], immunoglobulin transcripts (IGTs) [87], injury and rejection induced transcripts – intermediate (IRITD3) and – late (IRITD5) [93], injury-repair associated transcripts (IRRATs) [94], kidney transcripts (KT1, KT2) [95], mast cell-associated transcripts (MCATs) [96], quantitative CTL-associated transcripts (QCATs) [97], quantitative constitutive macrophage-associated transcripts (QCMATs) [86], ABMR+TCMR+all-rejection associated transcripts (RATs) [17], all-rejection associated transcripts (Rej-RATs) [85], TCMR-associated transcripts (TCMR-RATs) [85], and fibrillar collagen transcripts (FICOLs). Analyses will usually use selected PBTs from this list based on the specific focus of that analyses, e.g., rejection, injury, or inflammation. PBT scores can be calculated in different sets of biopsies as a way of comparing the disease processes present or absent in those populations, but PBTs can also assist in the interpretation of gene lists. For example, when top 20 differentially expressed genes are examined from a class comparison, each of those genes may be annotated as belonging to certain PBTs, helping to assign biologic or clinical meaning to the list as a whole.

**Table 3:** Commonly used pathogenesis-based transcript sets (PBTs)^*^

Transcript set	Abbreviation	Description of the transcripts
TCMR-related	QCAT	Cytotoxic T cell-associated^1^
ABMR-related	DSAST	DSA selective^2^
	NKB	NK cell transcript burden^3^
	ABMR-RAT^B^	ABMR selective^4^
	GRIT	Interferon gamma-inducible^5^
Increased after recent injury	IRRAT30	Injury-repair response associated^6^
	FICOL	Fibrillar collagen transcripts^7^
	IRITD3	Injury-repair induced transcripts, day 3^8^
	IRITD5	Injury-repair induced transcripts, day 5^8^
Atrophy-fibrosis	IGT	Immunoglobulin transcripts^9^

1. Hidalgo LG, Einecke G, Allanach K, Mengel M, Sis B, Mueller TF, et al. (2008) The transcriptome of human cytotoxic T cells: measuring the burden of CTL-associated transcripts in human kidney transplants.* Am. J. Transplant*. **8**(3), 637–646.

2. Hidalgo LG, Sis B, Sellares J, Campbell PM, Mengel M, Einecke G, et al. (2010) NK cell transcripts and NK cells in kidney biopsies from patients with donor-specific antibodies: evidence for NK cell involvement in antibody-mediated rejection. *Am. J. Transplant*. **10**(8), 1812–1822.

3. Hidalgo LG, Sellares J, Sis B, Mengel M, Chang J, Halloran PF. (2012) Interpreting NK cell transcripts versus T cell transcripts in renal transplant biopsies. *Am. J. Transplant*. **12**(5), 1180–1291.

4. Halloran PF, Potena L, Van Huyen JD, Bruneval P, Leone O, Kim DH, et al. (2017) Building a tissue-based molecular diagnostic system in heart transplant rejection: The Heart Molecular Microscope Diagnostic (MMDx) System. *J. Heart Lung Transplant*. **36**(11):1192–1200.

5. Halloran PF, Venner JM, Famulski KS. (2017) Comprehensive analysis of transcript changes associated with allograft rejection: combining universal and selective features. *Am. J. Transplant*. **17**(7), 1754–1769.

6. Famulski KS, de Freitas DG, Kreepala C, Chang J, Sellares J, Sis B, et al. (2012) Molecular phenotypes of acute kidney injury in human kidney transplants. *J. Am. Soc. Nephrol.* **23**(5), 948–958.

7. Famulski KS, Reeve J, de Freitas DG, Kreepala C, Chang J, Halloran PF. (2013) Kidney transplants with progressing chronic diseases express high levels of acute kidney injury transcripts. *Am. J. Transplant.* **13**(3), 634–644.

8. Famulski KS, Broderick G, Einecke G, et al. (2007) Transcriptome analysis reveals heterogeneity in the injury response of kidney transplants. *Am. J. Transplant.* **7**(11), 2483–2495.

9. Einecke G, Reeve J, Mengel M, Sis B, Bunnag S, Mueller TF, et al. (2008) Expression of B cell and immunoglobulin transcripts is a feature of inflammation in late allografts. *Am. J. Transplant.* **8**(7), 1434–1443. B. While ABMR-RATs are titled as ABMR-related, they are found in many instances of all-rejection.

*https://www.ualberta.ca/medicine/institutes-centres-groups/atagc/research/gene-lists.

## Risk scores, survival analyses, and outcome prediction in transplantation – MMDx and follow-up

Survival analyses require specific statistical methods in order to handle the complex style of data associated with examining the length of time from an initial point until either censoring or a designated event (events usually defined as graft failure in transplantation). Events do not occur in all individuals in a population, length of follow-up time varies between individuals, and survival data are rarely normally distributed, therefore survival analyses must take certain precautions to ensure that the conclusions about the population are valid.

### Censoring and events

‘Events’ can have many alternative definitions depending on what type of outcome analysis is desired. In some organs, time to patient death and time to graft failure are essentially identical (and common practice is to assume that these events are equivalent – for example, in heart or lung transplantation, patient death is usually a result of graft failure). In this case, ‘all-cause graft loss’ would be appropriate – that is, death from any reason would be considered an event. In other instances, patient death and graft failure are different events and should be carefully censored – for example, in kidney transplants a failure of the graft is usually the event of interest but will instead typically prompt a return to dialysis. This would require ’death-censoring’, considering graft failure an event, but any follow-up date without graft failure or patient death from any cause aside from graft failure would be censored, i.e., not an event.

### Biopsy-based survival predictions to understand risk of progression and failure

Survival predictions have challenges in that they are the prediction of a binary outcome (survival event, i.e., death or graft failure versus no survival event encountered), but the *actual* probability of graft or patient survival exists as a continuum between these two extremes [98]. It is possible to stratify the mean survival of high, medium, and low risk patients with reasonable precision, but predictive models have limitations including low discriminatory power at an individual (patient) level [98]. The differences between the survival of groups and individuals are not well understood [98], and while properly-generated predictive models can provide valuable insights into the survival probabilities of population groups, the differences between groups can be more meaningful [99].

While biopsy-based survival predictions per group – for example, groups split based on quintiles of scores or rejection diagnoses – will generate actuarial curves based on the real data, an individual survival curve only represents a predicted probability of survival at each time point post-biopsy. The use of individual patient probabilities at any given time point is also challenged because the relationship between continuous probabilities and the dichotomous truth of survival (i.e., either the graft is functioning or the patient has experienced graft failure) is unclear [98]. Even the best biopsy-based survival models are prone to sources of uncertainty: for example, sampling error or patient complications not included in the given model. Within MMDx survival/outcome analyses, we typically choose to show survival probabilities for a number of groups in the reference population, and the individual’s survival probability as compared to those groups. Our goal when providing survival prediction features in MMDx reports is to show the probability of future outcomes for that graft with as much precision as is possible using the tissue-based molecular measurements, while potentially informing the clinician in scenarios where closer management or increasing caution may be warranted [98].

Survival analyses have been useful in understanding risks across a population, and show important molecular mechanisms. Biopsy-based survival estimates indicate whether those organs with biopsies have been associated with increased risk of failure in the reference set of organs, but are not intended to be used as accurate predictors for the individual patient, and should be used in context of other measures of personal risk available to the clinician.

### Injury versus rejection features for predicting graft outcome post-biopsy

**Figure 7:**
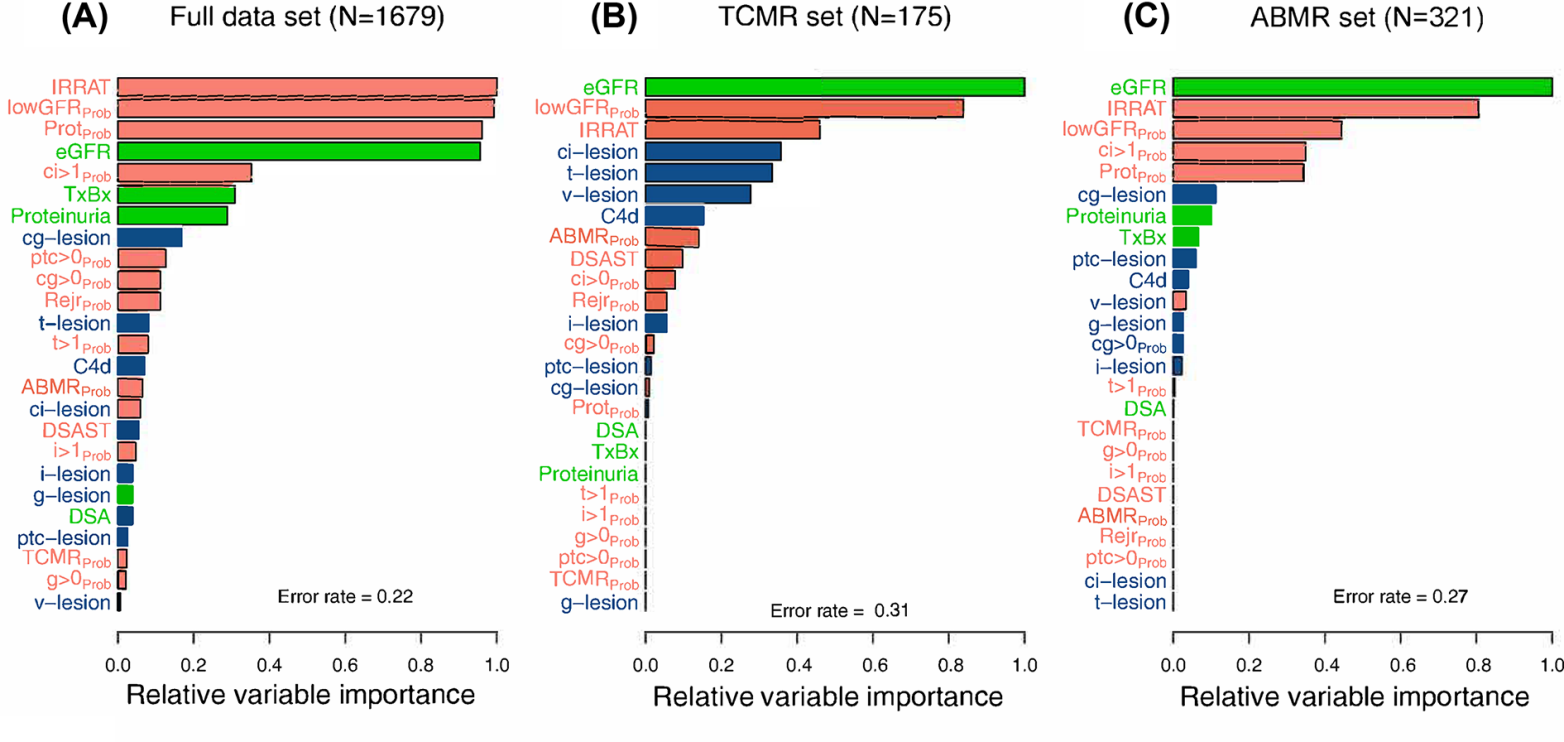
Various relationships between molecular, histological, and clinical variables and graft survival post-biopsy Relative variable importance in random survival forest analysis in (**A**) all biopsies (*N*=1679), (**B**) Biopsies with molecular TCMR (*N*=175), and (**C**) biopsies with molecular pure ABMR (*N*=321) [3,8]].

The relationship of molecular changes and kidney function to disease mechanisms such as rejection can help us to understand the pathophysiology of disease for example, the molecular changes in the renal tubule that underlie the tubulitis seen in TCMR [3,56]. Organ function is the principal determinant of outcomes in kidney and other transplants [3,8] (Figure 7). Our past studies have compared the relative variable importance between rejection and injury features, demonstrating that, when both rejection and injury features are available, injury features are ultimately the most important in predicting graft survival [3,8]. While some injury is undeniably a result of rejection episodes, there are many sources of potential parenchymal damage to the graft, and ultimately it is the extent and form of injury that determines probability of graft failure. MMDx reports have evolved over time to represent both rejection and injury features, taking into account the importance of understanding rejection states, but also the extent of tissue injury as it relates to risk of graft loss. In some cases, the rejection scores also add value (in addition to the injury scores) because the presence of rejection predicts that there will be new damage in the future.

## The interface between MMDx and the standard of care – other applications of MMDx algorithms

While the primary purpose of MMDx has been to define the molecular phenotypes of rejection and injury in organ transplants, some MMDx algorithms and diagnoses have been used to learn more about standard of care features in the transplant population. In this way, diagnostic systems can each provide independent estimates of graft rejection, injury, and prognostic features, allowing for comparison of diagnostic discrepancies between systems, potential clarification of ambiguous clinical cases, and further development of our overall understanding of clinical phenotypes.

### The AutoBanff algorithm – decision trees to study histology discrepancies and access to a diagnosis strictly following Banff guidelines

**Figure 8:**
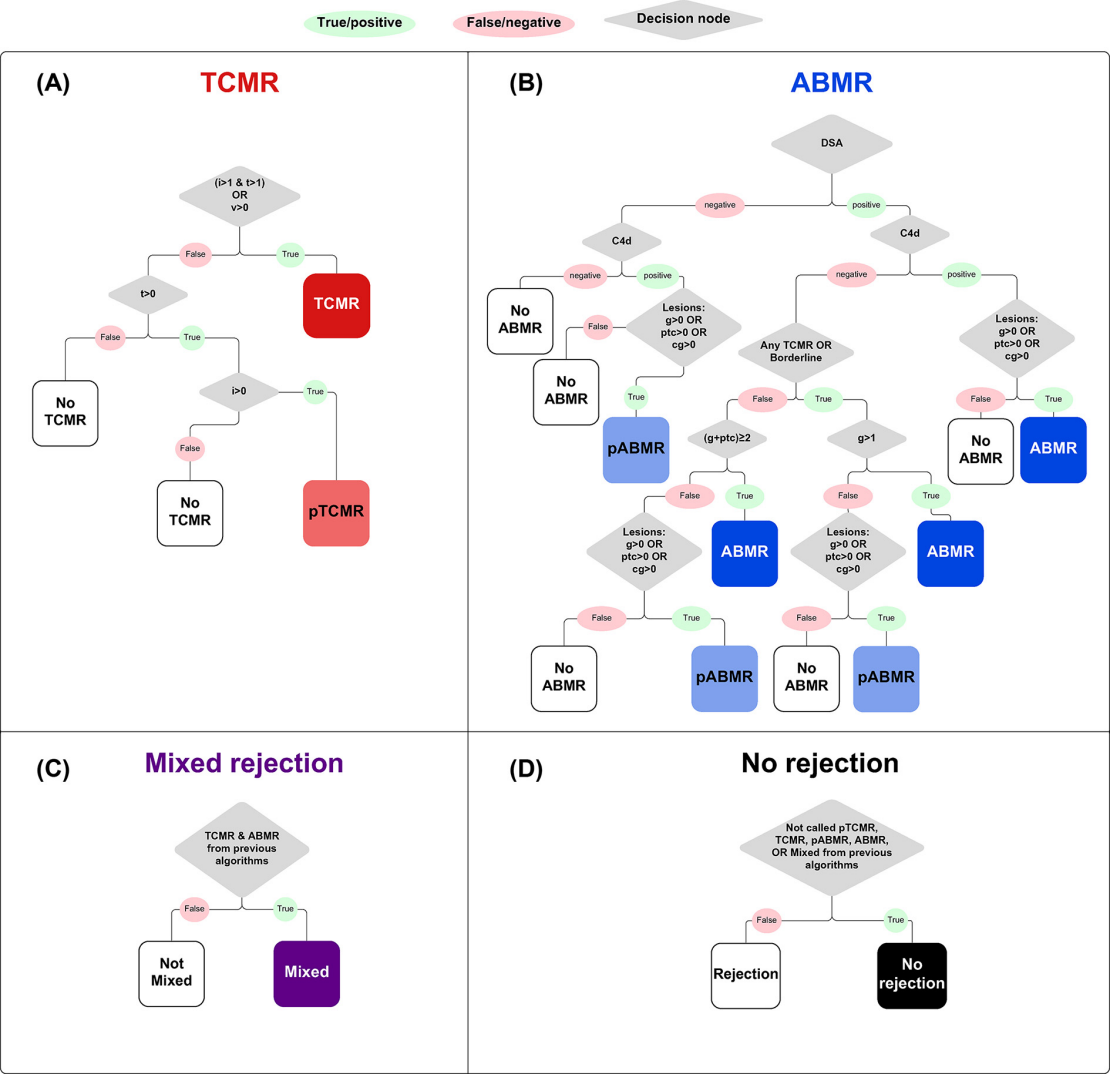
The algorithm for assigning AutoBanff diagnoses using the eight canonical rejection lesion scores assigned by the local center pathologists. Algorithm was based on the most recent iteration of the Banff guidelines [100]. The algorithm works by elimination, and moves through panels (**A–D**) in that order.

In some previous analyses, we have used diagnoses assigned using an ‘AutoBanff’ algorithm, a decision tree-style algorithm originally developed in 2018 and updated in 2022, and designed to apply Banff guidelines to lesion scores as reported by the local center and assign a final diagnosis. The algorithm for AutoBanff was developed in the R computing language and applied to a population of 1679 biopsies [1,10] (Figure 8). This algorithm was based on Banff 2019 guidelines [100] converted to a ‘decision tree’ format, where a series of subsequent decisions based on data results in a final diagnosis. The algorithm functions with a decision-making hierarchy; in the order A→B→C→D to allow for the diagnosis of some types of rejection that would rely on a previous decision (i.e., a diagnosis of Mixed rejection requires prior knowledge about the presence or absence of TCMR and ABMR in the biopsy). In short, AutoBanff is a programmed algorithm built to strictly apply Banff 2019 guidelines to eight canonical rejection features recorded for each biopsy, as a simulation of a single expert pathologist strictly applying Banff guidelines. AutoBanff generates one of six different diagnoses: ABMR, possible ABMR (pABMR), TCMR, possible TCMR (pTCMR), Mixed Rejection, and No rejection (NR).

Some factors were not available or not used for the AutoBanff algorithm, i.e., thrombotic microangiopathy or other aspects of guidelines that do not constitute a clear branching decision point for all cases. Overall, these conditions were rare in the population and did not have a substantial effect on the accuracy of the algorithm (e.g., thrombotic microangiopathy occurred in only 21/1679 cases when AutoBanff was originally developed).

While the AutoBanff algorithm has provided an unbiased, histology-based diagnosis that strictly follows Banff guidelines (and provides a simulation of semi-central review based on the locally-assessed lesions, when needed), it has also indicated that additional clinical information outside of the features considered by Banff guidelines and/or professional judgment provided by the expert pathologist adds value with regards to agreement with MMDx, i.e., the locally reported histology diagnoses from each center (translated into six categories for accurate comparison) agrees more with the MMDx molecular diagnosis than the AutoBanff diagnosis [101]. Essentially, Banff provides guidelines, not commandments: pathologists actually do not follow the Banff guidelines strictly in all cases and instead use their professional judgment. This leads to *increased* agreement with MMDx, i.e., expert pathologists’ judgement adds value. Additionally, this suggests that some Banff guidelines may not be as closely tied to the true clinical phenotypes as we would assume, and revisiting the guidelines in the context of the current literature landscape might be advisable.

Additionally, AutoBanff has provided us in some analyses with a simulated ‘central pathology review’ of biopsies, using the lesions and features as reported by the local center [27]. These findings show how comparisons of independent diagnostic systems can help refine our understanding of the guidelines themselves, and the clinical phenotypes they describe. AutoBanff also allows us to simulate whether certain changes in Banff rules would allow better agreement with external measurements such as dd-cfDNA or MMDx.

### Regression equations – training algorithms on MMDx diagnoses for enhancing histologic diagnoses and using independent biopsy assessment systems to refine clinical diagnoses as a whole

We revisit the concepts of ‘step 1’ and ‘step 2’ (see Introduction), for establishing the probability that a disease is present: step 1, measure designated features; and step 2, interpret feature measurements using predefined algorithms [5]. MMDx rejection diagnoses can be used to train step 1 histology feature-based regression models, and also assess the relative importance of each histology step 1 feature because the molecular calls are independent of histology.

**Figure 9:**
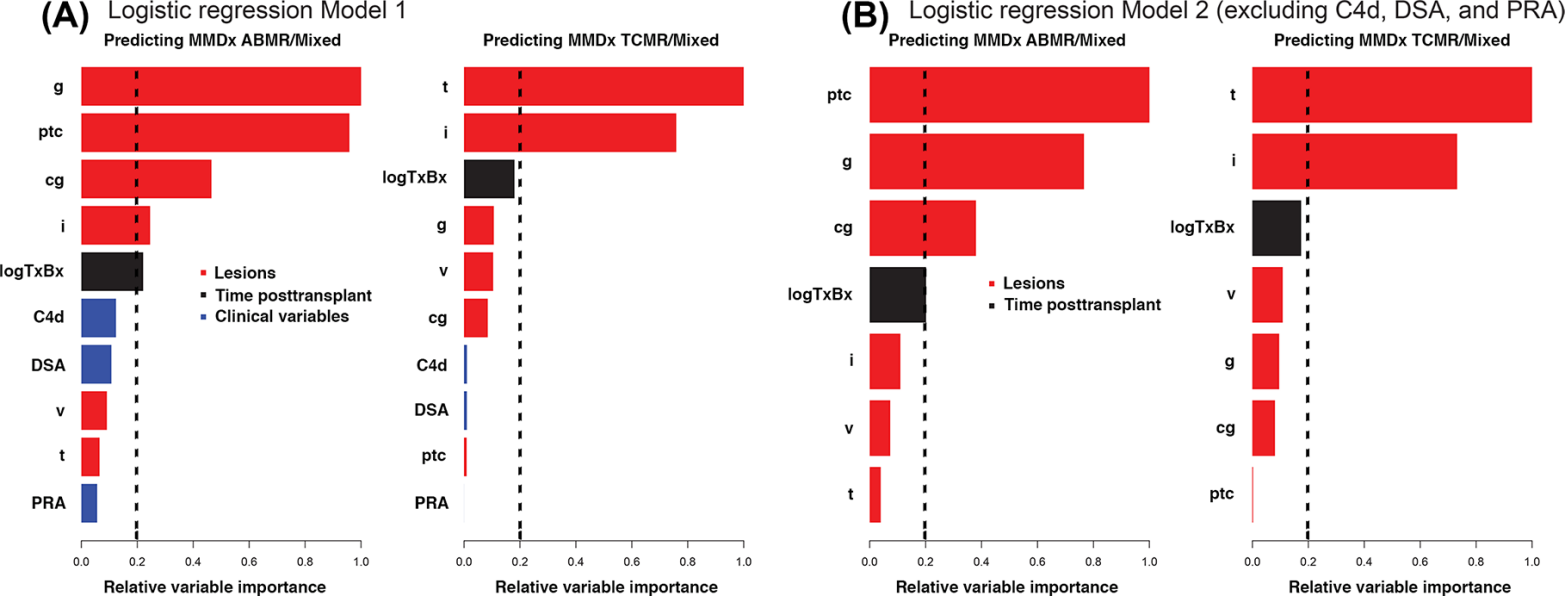
Logistic regression models (Model 1 and 2) predicting MMDx ABMR/Mixed or TCMR/Mixed in kidney transplants (*N*=1679) Bar plots show the relative importance of variables in the prediction of rejection using logistic regression, (**A**) with and (**B**) without using C4d, PRA and DSA. Vertical dashed lines designate an arbitrary cutoff for variable significance [5].

We generated models from Banff diagnoses (as a single 4-level factor: ABMR, TCMR, Mixed, or NR) in addition to the step 1 individual features (histologic g-, ptc-, cg-, v-, i-, and t-lesion scores, plus time posttransplant, and binary definitions of C4d, DSA, and PRA) for the prediction of MMDx ABMR and TCMR signouts [5]. Regression models were used to show the relative variable importance of step 1 rejection features for predicting ABMR and TCMR (Figure 9) [5]. For predicting MMDx ABMR, the most important features were ptc- and g-lesions, while cg-lesions were moderately important and v-lesions were not important (Figure 9A) [5]. For predicting MMDx TCMR (Figure 9B), t-lesions were most important, while i-lesions and time posttransplant were also important and v-lesions were again unimportant [5]. Error rates were consistent regardless of if C4d or DSA were included in the model. These findings showed how MMDx signouts can give insight into the relationship between various histologic and clinical features versus the disease phenotype, and findings were consistent with previous studies showing that some lesions (i.e., v-lesions [102]) are not as closely associated with the clinical phenotype as we once assumed.

To establish the potential utility of adding lesion-based regression scores to the Banff system, we examined the potential impact of using regression assessments on 887 biopsies that Banff histology uniformly called No rejection [5]. Banff No rejection biopsies identified as TCMR, ABMR, or Mixed rejection by regression models had lesions expected for those rejection states but *also* had molecular abnormalities characteristic of the predicted rejection state (Table 4) [5]. Banff No rejection biopsies with regression-assigned ABMR diagnoses had higher mean ABMR-associated lesion scores (g-, ptc-, and cg-lesions) and were more likely to be DSA-positive than those with low ABMR regression scores [5]. Similarly, we found that high TCMR regression scores predicted high i- or t-lesions in the biopsy [5]. More importantly, in both cases we found that relevant rejection-associated molecular features were also increased when regression scores increased: Banff No rejection biopsies that regression called ABMR had high ABMR-related molecular scores (e.g., ABMR_Prob_, g>0_Prob_ classifier score) and the cases regression called TCMR had higher TCMR-related molecular scores (e.g., TCMR_Prob_, t>1_Prob_ classifier) [5]. These results showed that regression equations trained on MMDx calls may assist the clinician in identifying potentially at-risk cases that warrant additional follow-up.

**Table 4:** Effect of considering Model 2 regression scores on interpretation of biopsies with no Banff histology rejection (*N*=887)^1^

Recorded histology and molecular features in 887 biopsies with no rejection by Banff guidelines		Regression diagnoses			
		No rejection-regression (*N*=827)	TCMR-regression (*N*=2)	Mixed-regression (*N*=3)	ABMR-regression (*N*=55)
**Histology lesion scores, plus DSA and C4d**					
**TCMR-related**	t (tubulitis)	0.11	**2.00** ^4^	**2.67** ^4^	**0.23** ^2^
	i (interstitial infiltrate)	0.22	**2.00** ^4^	**3.00** ^4^	**0.73** ^4^
**All rejection-related**	v (vasculitis)	0.00	0.00	0.00	**0.04** ^4^
**ABMR-related**	g (glomerulitis)	0.11	0.00	0.50	**1.51** ^4^
	ptc (capillaritis)	0.08	0.00	**3.00** ^c^	**1.04** ^4^
	cg (double contours)	0.05	0.00	0.00	**1.02** ^4^
**Atrophy-fibrosis-related**	ci (scarring)	1.06	**2.50** ^2^	1.67	**1.51** ^4^
	ct (atrophy)	1.00	2.50	1.67	1.27
**DSA-related**	DSA positivity	0.35	0.00	0.00	0.32
**C4d-related**	C4d positivity	0.06	—	**0.50** ^2^	0.14
**Transcript set and molecular classifier scores**					
**TCMR-related classifiers**	TCMR classifier (TCMR_Prob_)	0.04	**0.21** ^2^	**0.223**	**0.042**
**All-rejection-related**	Rejection classifier (Rej_Prob_)	0.15	0.33	**0.61** ^2^	**0.45** ^4^
	IFNG-inducible (GRIT3)	0.39	**0.87** ^2^	**1.18** ^3^	**0.65** ^4^
**ABMR-related**	DSA-selective (DSAST)	0.09	0.04	0.20	**0.39** ^4^
	NK cell burden (NKB)	0.40	0.60	**0.83** ^b^	**0.87** ^4^
	ABMR classifier (ABMR_Prob_)	0.10	0.06	0.08	**0.32** ^4^

1 Sikosana MLN, Reeve J, K.S. M-T, Halloran PF, Investigators I. Using regression equations to enhance interpretation of histology lesions of kidney transplant rejection. Transplantation. 2023;108(2):445-54. Wilcoxon test compared with the no rejection group (significant values bolded)

2 *P* value <0.05.

3 *P* value <0.01.

4 *P* value <0.001.

**Figure 10:**
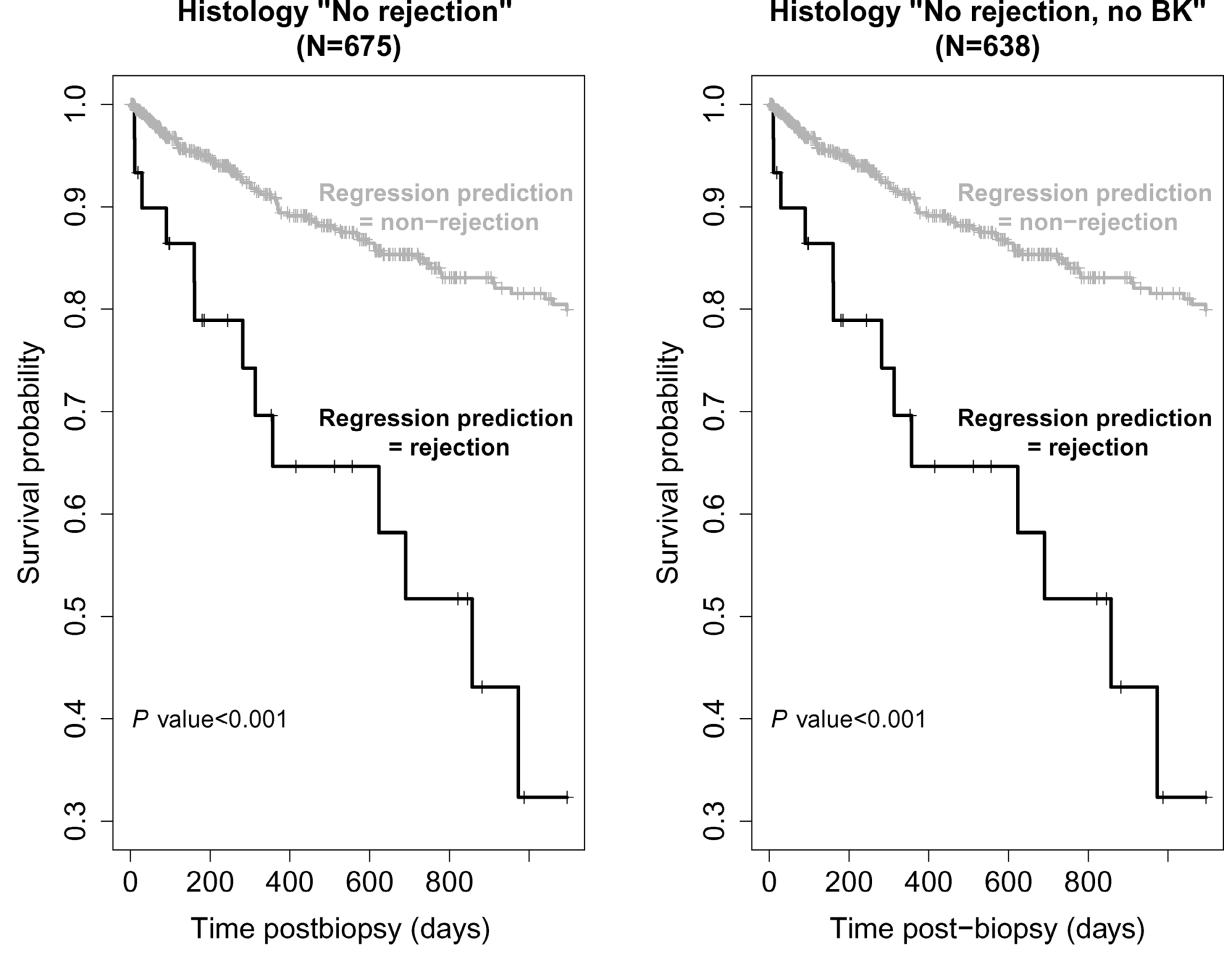
Survival curves Survival three years post biopsy in biopsies called histologic No rejection, split by whether they are called rejection by the regression Model 2 (all histologic No rejection samples, *N*=675) [5].

We assessed the impact on survival curves when combining regression rejection diagnoses in Banff No rejection biopsies [5]. The 3-year graft survival of Banff No rejection samples (*N*=638) split by their regression model rejection status is shown in Figure 10 [5]. There were significantly more graft losses within the Banff No rejection cases that the regression models identified as rejection (*P*<0.001). We found that survival in the regression-based non-rejection group was significantly better than in the rejection group regardless of which random sample per transplant was used.

Overall, these regression models may improve our clinical management by noting cases with increased risk that have been missed by current guidelines, allowing for earlier interpretation when other binary elements (e.g., DSA status) are missing, and flagging potentially concerning cases for the clinician and the pathologist.

## Concluding thoughts – Lessons from the MMDx project

The initial hypothesis driving these genome-wide analyses was that MMDx would reveal molecular mechanisms underlying the disease states in organ transplants, and could provide a new diagnostic system that could meet the need for precision and accuracy in biopsy assessment from a small amount of tissue. We hoped that the lessons from MMDx-Kidney could be extrapolated to other organ transplants and even to other areas of medicine. This hypothesis was proven to be true throughout the course of MMDx studies and across many biopsy populations. This approach has developed an independent diagnostic system, rigorously calibrated with special care to avoid over- or under-fitting, that can be used to recalibrate other diagnostic systems. The MMDx approach can be followed to generate other new biopsy diagnostic systems that are robust and highly reproducible, and can clarify many ambiguous or challenging cases where the standard of care is insufficient. The ultimate goal is better outcomes, and making more accurate disease assessments is the starting point for better use of existing treatments and better trials to target the disease states that prevent long term organ health.

## Data Availability

Data for published work is available on the Gene Expression Omnibus.

## Abbreviations

AMAT: alternative macrophage-associated transcript
BAT: B cell-associated transcript
cIRIT: cardiac injury and repair-induced transcript
DAMP: damage-associated molecular pattern-associated transcript
eDSAST: endothelial DSA-selective transcript
ENDAT: endothelial cell-associated transcript
FFPE: formalin-fixed paraffin-embedded
FICOL: fibrillar collagen transcript
GRIT: γ-interferon and rejection associated transcript
IGT: immunoglobulin transcript
IRRAT: injury-repair associated transcript
KT: kidney transcript
MCAT: mast cell-associated transcript
MMDx: Molecular Microscope® Diagnostic System
QCMAT: quantitative constitutive macrophage-associated transcript
RAT: rejection associated transcript
